# Multi-biomarker panel signature as the key to diagnosis of ovarian cancer

**DOI:** 10.1016/j.heliyon.2019.e02826

**Published:** 2019-12-05

**Authors:** Thingreila Muinao, Hari Prasanna Deka Boruah, Mintu Pal

**Affiliations:** a Biotechnology Group, Biological Sciences and Technology Division, CSIR-North East Institute of Science and Technology, Jorhat, Assam, 785006, India; b Academy of Scientific and Innovative Research, CSIR-North East Institute of Science and Technology, Jorhat, Assam, 785006, India

**Keywords:** Cancer research, Multi-biomarker signature, Ovarian cancer, Early detection

## Abstract

Early detection of ovarian cancer has been a challenge to manage the high mortality rate caused by this deadly disease. The trends in mortality have been reduced by the scientific contributions from the corners across the globe, however accounting for the fifth leading cause of gynecological mortality. The complexities in the clinical presentation, origin of tumor, and gene expression profiles had added to much difficulty in understanding and diagnosis of the disease. Stage 1 diagnosis of ovarian cancer improves the 5-year survival rate to around 92%. Cancer antigen-125 (CA-125) is the gold standard tumor marker found at abnormally high levels in the blood of many women in ovarian cancer. However, many non-cancerous conditions exhibit high levels of CA-125 and several women have normal CA-125 level in the early stage of ovarian cancer, suggesting CA-125 biomarker is not specific enough for the screening of early stage ovarian cancer. In addition, several other biomarkers, including HE4 have been added in the diagnostic field for higher sensitivity and specificity in the diagnosis and progression of ovarian cancer. HE4 is a prospective single serum biomarker which has been approved by the FDA to monitor the disease progression in epithelial ovarian cancer. However, owing to low sensitivity and specificity, combination of a panel of biomarkers has been proposed in the diagnosis of the disease. Based on extensive biomarkers research findings, here we discuss current trends in diagnostic approaches and updated potential several panels of cancer biomarkers for early detection of ovarian cancer. It has been recently reported that CA125 in combinations with two or more biomarkers have outperformed single biomarker assays for early detection of the disease. Moreover, CA-125 with CA 19–9, EGFR, G-CSF, Eotaxin, IL-2R, cVCAM, MIF improved the sensitivity with 98.2 % and specificity of 98.7% in early stage detection of ovarian cancer. Overall, this review demonstrates a panel of biomarkers signature as the potential tool for prototype development in future and other advanced approaches for early diagnosis of ovarian cancer to avoid false-diagnosis and excessive cost.

## Teaser

Cell Death; Cell Differentiation; Biomolecules; Chemotherapy; Oncology; Toxicology, Multi-biomarker signature; ovarian cancer; Early detection

## 1 Introduction

Ovarian cancer does not occur frequently, nevertheless accounts for the fifth-leading cause of cancer mortality among women worldwide. According to the data from 2013-2015, about 1.3 percent of women are expected to be diagnosed with ovarian cancer during their lifetime. In addition, 2011–2015 data report 11.6 per 100,000 annual cases of women and 7.2 per 100,000 deaths per year with ovarian cancer (from https://seer.cancer.gov/statfacts/html/ovary.html; SEER, Surveillance, Epidemiology, and End Results Program). Such poor projection of this disease is due to its advance metastasis at the time of presentation and difficulty in diagnosis in its early stage. More than 60 % of cases are diagnosed after the cancer has metastasized. The five year survival estimate of ovarian cancer when the disease is localised in the ovary at the time of diagnosis is 92 %; however, diagnosis at late stage dropped the estimate to 29 % (Siegel et al., 2017). In fact only 25 % of ovarian tumors are identified at stage I (Badgwell and Bast, 2007). Therefore, the strategy to manage this disease is to identify a biomarker(s) that could diagnose ovarian cancer at the early stage with high specificity and sensitivity. The challenges remained not only in such biomarker identification but the complexities in the characteristics of the disease itself in terms of its epidemiology, histopathology, or genetic features that contribute in the poor understanding of the disease (Muinao et al., 2018a). Ovarian cancer is viewed as a heterogeneous disease comprising of several types and subtypes (Kobel et al., 2008; Ayhan et al., 2009). The extra-ovarian tissue originations of the epithelial ovarian cancer contribute in the intricacies of the disease.

Ovarian cancers are broadly categorized into germ cells, sex cord-stromal cells and epithelial cells types, where, epithelial ovarian cancer constitutes above 95 % of the disease (Quirk and Natarajan, 2005; Muinao et al., 2018b). Histological perspective of ovarian cancer further outlined four transcriptional subtypes of epithelial ovarian cancer as serous, endometrioid, mucinous, and clear cell ovarian cancer of which serous tumors are the most common, representing 40 % of all epithelial tumors (Quirk and Natarajan, 2005; Muinao et al., 2018b). Another classification identified ovarian cancer as the low grade type I with frequent mutations in *BRAF*, *KRAS* (Quirk and Natarajan, 2005), and *PTEN;* whereas high grade type II that harbour mutations in *p53* (Banerjee and Kaye, 2013), *BRCA1*, and *BRCA2*. The growth of low grade subtype is more indolent, thereby increasing the possibility of early stage diagnosis. On the contrary, high grade type II subtypes are more rapid in their growth and hence difficult for early detection. The identification of biomarkers of ovarian cancer has not been successful for the diagnosis of ovarian cancer let alone the screening with each individual types and subtypes associated biomarkers. Studies have however reported several potential biomarkers for detection of early stage ovarian cancer. This review attempts to draw the importance of the combination of biomarker panels rather than a single biomarker approach in the early diagnosis of ovarian cancer for higher specificity and sensitivity.

## 2 Trends in the diagnostic approaches

Multivariate index assay has been developed in determining the possibility of ovarian cancer as a triage to ovarian masses and diagnostic aid. Jacobs and group proposed an algorithm of “Risk of Malignancy Index” (RMI), where CA125 is combined with ultrasound and menopausal status (Jacobs et al., 1990). Three types of RMI versions, I, II and III, have been used in different countries for clinical practice (Le et al., 2009; Clarke et al., 2009) for prediction of ovarian cancer in women with pelvic mass. Sensitivities for this prediction showed 78 %, 79 %, and 74 %, and corresponding specificities of 87 %, 81 %, 91 %, for RMI I, II, and III respectively (Jacobs et al., 1990; Geomini et al., 2009; Tingulstad et al., 1996).

Ova1 is a multivariate index assay approved by FDA comprising of 5-serum protein biomarker panel for the triage of patients with pelvic mass (Uelandet al.2011, Zhang and Chan, 2010) for low or high risk ovarian cancer. It comprised of second generation CA125-II, transferrin, beta-2 microglobulin, apolipoprotein A-1, and transthyretin. Ova1 performance score produced 96 % sensitivity at 35 % specificity in 590 women slated for resection of ovarian tumor (Ueland et al., 2011; Miller et al., 2011). Next in 2009, Risk of Ovarian Malignancy Algorithm (ROMA) was designed by Moore and group for predicting epithelial ovarian cancer in women affected with pelvic mass (Moore et al., 2009). ROMA is a combination of HE4, CA125 and menopausal status that set the specificity at 75 % to determine the sensitivity. However, there has been a contradictory effect in the diagnostic and predictive application of this algorithm.

FDA approved the next generation of Ova1, the Overa in 2016; exhibited 91 % sensitivity and 69 % specificity (Ueland et al., 2011). It combined CA125-II, HE4, apolipoprotein A-1, follicle stimulating hormone and transferrin (Coleman et al., 2016, Kumari, 2018). Another biomarker-based index named Copenhagen Index (CPH–I) was developed by Karlsen et al. that performed similar to ROMA and RMI, however without considering ultrasound and menopausal status. It is a combination of HE4, CA125 and age (Karlsen et al., 2015). Importantly, these tests are not ideal diagnostic tests, but rather triage or referral tests. Females with ovarian tumor when summoned for surgery, these triage tests are performed to determine the possibility of malignancy. This is significant for the primary care provider as a determinant for referring to a gynecologic oncologist.

## 3 Present diagnostic biomarkers of early-stage ovarian cancer

Over 70 % of ovarian cancers are diagnosed at advanced stage (Holschneider and Berek, 2000). The challenge is to identify the biomarkers for the early detection of ovarian cancer that could benefit in clinical output. Owing to the rare occurrence of ovarian cancer, maximum specificity and sensitivity should be the target for screening of early stage disease (Menon and Jacobs, 2001; Jacobs and Menon, 2004) achieving 99.6 % of specificity and >75 % sensitivity to overcome insupportable false-positive results and thereby achieve a positive predictive value of 10 % (Yang et al., 2017). CA-125 is one of the most commonly used serum biomarker in the diagnosis of ovarian cancer. However, significant increase in the level of CA 125 was found in adenomyosis, uterine myoma, endometrial pathology, and endometriosis of the ovary (Kim et al., 2019). Moreover, CA125 is not only increased in about 80 % of ovarian cancer but also 50 % rises are observed in stage I epithelial ovarian cancers (Patriotis et al., 2017; Zurawski et al., 1988). Therefore, using CA125 as the only biomarker for diagnosis will miss out those which do not express this antigen. Circulating concentrations of CA-125, HE4, prolactin, IL-2R, CA 15–3, CA 19–9, CA 72–4, MIF, Cyfra 21–1, TNFR1, TNFR2, IL-6, IL-7, IL-10, IGFBP1, TSH, TNF-α, GH, TIMP-1, ACTH, and osteopontin were reported to be significantly (*P* < 0.001) higher in serum of patients with early-stage ovarian cancer compared to healthy women, while serum levels of HE4, IL-2R, prolactin, CA 15–3, CA 19–9, CA 72–4, Cyfra 21–1, TNFR1, TNFR2, IL-6, IL-7, IL-10, TNF-α, TSH, IGFBP1, MMP-7, VCAM-1, eotaxin-1, FSH, LH, ErbB2, ApoA1,TTR, adiponectin, and CD40L differed significantly (*P* < 0.01) between patients with early-stage (stages I and II) and late-stage (stages III and IV) ovarian cancer (Yurkovetsky et al., 2010). However, serum biomarkers other than CA125 are not currently used as a detection tool for early stage disease due to their lower sensitivity or specificity (Baron et al., 2003; Perkins et al., 2003) and therefore the hunt is on early biomarkers with high specificity and sensitivity continues to predict the occurrence of metastasis before it manifests in the patients. Considering the existing current studies based on the sensitivity and specificity, several potential biomarkers for early diagnosis of ovarian cancer have been summarized as listed in Tables 1 and 2.

**Table 1:** List of potential single biomarkers for early detection of ovarian cancer.

Biomarker	Sensitivity	Specificity	Methods performed	References
CA-125	92%	80%	Electrochemilluminescence (ECLIA) technique	Lycke et al., (2018)
HE4	71.19 %	85.0%	Clinical chemistry and immunochemistry	Zhang et al., (2019)
Osteopontin	7.6%	98%	ELISA	Moore et al., (2008)
VEGF	74%	71%	ELISA	Cooper et al., (2002)
KLK6	21%–26%	95%	Immunoassay	Diamandis et al., (2003)
IL-6	84.1%	86%	LabMAP assays	Gorelik et al., (2005)
IL-8	65.5%	98%	LapMAP technology	Lokshin et al., (2006a)
Transthyretin	47%	95%	Singleplex Luminex bead assays	Cramer et al., (2011)
Prostasin	51.4%	94%	Enzyme-linked immunosorbent assay	Mok et al., (2001)
SMRP	15.4%	98%	MESOMARK™ Assay	Moore et al., (2008)

**Table 2:** Panels of biomarkers for early stage detection of ovarian cancer.

Biomarkers	Sensitivity	Specificity	Methods	Reference
Apolipoprotein A1 (APOA1) and transthyretin	52.4%	96.5%	SELDI-TOF-MS Protein Chip array chromatographic assay	Moore et al., (2006)
HE4 + CA125	45.9%	95%	MESOMARK™ Assay	Moore et al., (2008)
human kallikrein 6 (hK6) + CA125	42%	90%	Immunoassay	Diamandis et al., (2003)
Transthyretin with CA-125, ApoA1 and transferrin	96%	98%	Chemiluminescence and immunoturbimetry technology	Nosov et al., (2009)
CA-125, HE4, CEA, and VCAM-1	86%	98%	Bead-based xMAP immunoassays	Yurkovetsky et al., (2010)
CA125, HE4, E-CAD, and IL-6	84.2%	95.7%	Simple Plex™ immunoassay	Han et al., (2018)
Osteopontin, leptin, prolactin and insulin-like growth factor-II (IGF-II)	95%	95%	Microarray analysis and ELISA assays	Mor et al., (2005)
Osteopontin, leptin, prolactin, insulin-like growth factor-II (IGF-II), macrophage inhibitory factor (MIF) and CA-125	95.3%	99.4%	ELISA	Kim et al., (2009)
**CA 125, CA 19–9, EGFR, G-CSF, Eotaxin, IL-2R, cVCAM and MIF**	**98.2%**	**98.7%**	**LapMAP™ technology**	**Lokshin et al. (2006b)**
Transthyretin, CA-125, ApoA1 and connective tissue-activating protein III	84%	98%	Immunoassay, SELDI-TOF-MS, Protein Chip arrays	Clarke et al., (2011)
CA-125, transferrin, TTR + ApoA1	89%	92%	Chemiluminescence technology and ​immunoturbimetry technology	Su et al., (2007)
VEGF + CA-125 + HE4	84%	82%	ELISA and chemiluminescent microparticle immunoassay	Lawicki et al., (2013)
ApoA1 + CA-125 + TTR	93.9%	95%	multiplex liquid assay system	Kim et al., (2012)
β2- microglobulin (β2-M), ApoA1 and CA-125	94%	98%	Multiplexed fluorescence spectroscopic, and Surface Plasmon Resonance spectroscopy	Pal et al., (2015)
CA-125, CA 72–4, CA 15-3, and M-CSF	68%	98%	Radioimmunoassay	Skates et al., (2004)
CA-125, apolipoprotein A1, truncated form of transthyretin, and a cleavage fragment of inter–alpha-trypsin inhibitor heavy chain H4	74%	97%	Immunoassay	Zhang et al., (2004)

Nevertheless, none of the single biomarker for the detection of early stage ovarian cancer has achieved the required specificity and sensitivity. Several studies based on multibiomarker approaches have been reported to improve the sensitivity over the single biomarker at similar specificity in diagnosing early stage ovarian cancer. CA125 in combinations with several other serum tumor biomarkers have been tested. For example, CA-125 and HE4 were shown to be the best among all two biomarker combinations in distinguishing the benign cells from early stage of ovarian cancer at 74.2 % sensitivity and 85 % specificity, whereas, CA-125, HE4, and EGFR considerably distinguish the benign from malignancy at 75.9 % sensitivity and 87.5 % specificity (Nolen et al., 2010). In another instance, a panel of six biomarkers consisting of CA-125, osteopontin, leptin, prolactin, MIF and IGF-II improved the sensitivity at 95.3 % and specificity at 99.4 % for ovarian cancer detection (Visintin et al., 2008). However, another study reported that a panel of four-biomarkers for early stage ovarian cancer including CA125 in combinations with 16 other biomarkers could achieve a better sensitivity ranging from 92.6% to 96.1% at 98% specificity in comparison to either two or three biomarker panels, when examined using MMC algorithm. In this study, CA-125, HE4, CEA, and VCAM-1 demonstrated as the highest diagnostic biomarker combination for early stage ovarian cancer achieving the sensitivity of 86 % and specificity of 98 % when validated in a training set. In addition, this four-biomarker panel were able to produce a comparable classification for the four most common histologic types of epithelial ovarian cancer at 85 %–90 % sensitivity (Yurkovetsky et al., 2010).

**Fig. 1:**
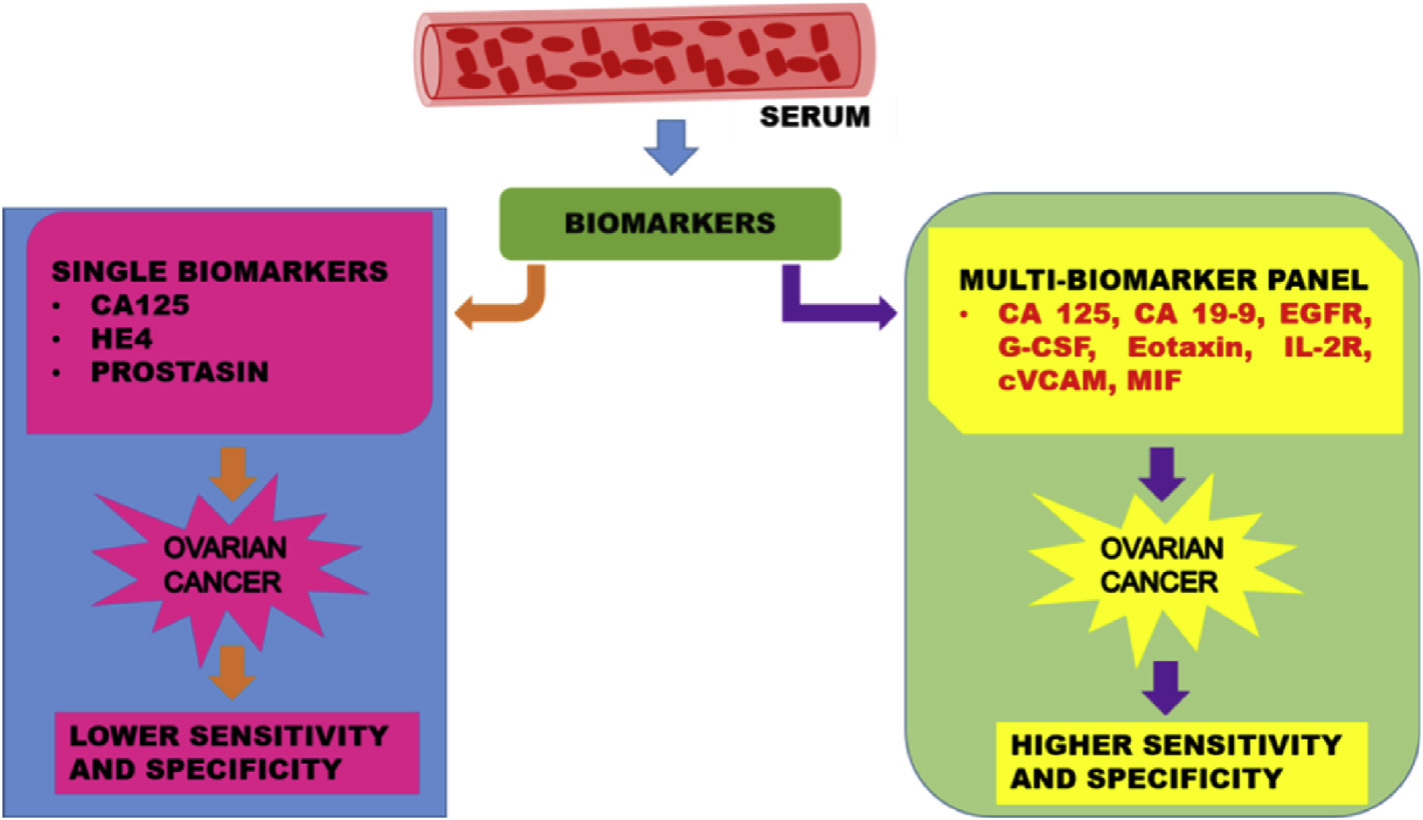
Schematic diagram represents the key multi-biomarkers panel as effective screening tool for early detection of ovarian cancer with high sensitivity and specificity.

Overall, recent studies reported the best performance of CA-125 with CA 19–9, EGFR, G-CSF, Eotaxin, IL-2R, cVCAM, MIF that improved the sensitivity with 98.2 % and sensitivity of 98.7 % in diagnosing the early stage ovarian cancer as highlighted in Table 2. Here, we propose that multi-biomarker panel is a better version for the early detection of ovarian cancer. The representation of the comparison of single biomarker and the multi-biomarkers with respect to the healthy controls based on sensitivity and specificity is shown in Fig. 1.

## 4 Other advanced approaches for early diagnosis of ovarian cancer

Several evidences have highlighted a large number of biomarkers with potential utility for detection of early ovarian cancer; however, the feasibility of its application in clinical settings has been a challenge. Hence, the combination of some biomarkers has not worked as a better alternative than the single biomarker to give high sensitivity and specificity. For instance, differentiating the benign from malignant cases of ovarian cancer by ROMA has produced an AUC of 91.2 %. However, further addition of YKL-40, transthyretin, ApoA1, Beta 2 microglobulin, transferrin, and LPA to ROMA has however not achieved remarkably better AUC (Moore et al., 2019). Therefore, careful selection of algorithm and meticulous scientific approach will improve the efficacy of biomarker combination and prediction of early stage ovarian cancer.

### 4.1 Mathematical and statistical algorithm

While the present diagnostic tool of ovarian cancer checks on the level of CA-125, it produces several false positive results. The level of basal biomarkers that show substantial changes at an instant (change-point) prior to the increase in CA125 that could have been an important determining factor in identifying the early disease are commonly missed out leading to higher chances of diagnosis when the disease has advanced. Mathematical algorithm such as the hierarchical Bayesian Model and Markov Chain Monte Carlo methods have been adopted in this area to estimate the change-point of biomarkers in cases before or those without showing the change point in CA125 and tried to fill the gap. The findings showed that ovarian cancer biomarkers such as HE4 and glycodelin exhibited a change-point in 80 % and 60 % respectively; whereas CA125 level does not show change-point. Moreover, the change point of these two biomarkers displayed a higher probability of occurring earlier than CA125 (Marino et al., 2017). The study summarizes in favour of the combination of CA125 with HE4 or glycodelin rather than CA125 alone as a biomarker to improve the efficiency for early diagnosis of ovarian cancer. When considering a biomarker panel, simply combinations of biomarkers in a group is not adequate but choosing the right combination matters and remains a challenge. A theoretical finding based on multi-variant normal distribution and linear algorithm depicted that addition of a marker, though of low predictive potential, to a primary marker will increase AUC if the additional marker is negatively correlated with the primary marker. On the contrary, if the additional marker is positively correlated with the primary marker, then the possibility to increase the AUC is less even if the additional marker has high predictive potential on its own. For example, Prolactin with relatively lower AUC on its own, however, owing to its negative correlation with CA125, display a higher AUC in combination with CA125 compared to biomarkers with higher univariant AUC than prolactin that has positive correlation with CA125 (Pinsky and Zhu, 2011). Hence it is difficult to find the best biomarker combinations in predicting the disease and therefore a statistical method might be helpful in drawing the best possible biomarker combination. Further, a two-step multi-biomarkers combination for possible use in clinical screening has been reported. In this, the first assay detects the expression of a biomarker or a panel of biomarkers to streamline the disease enriched population and minimizes the true negatives. Secondly, the expression of the second biomarker or biomarkers panel is detected to identify more precisely the true positive results. Here, HE4 at level greater than 1.8 ng/ml is considered positive in the first step. In the second step, Receiver Operating Characteristic (ROC) curve Best cutoff algorithm considered the outcome positive if CA125 is positive at >35 U/ml, or if any two of Glycodelin, MUC-1 or Plau-R are tested positive. This algorithm detects the early stage ovarian cancer at sensitivity and specificity of 76.7 % and 97.2 % respectively (Havrilesky et al., 2008).

### 4.2 Raman spectroscopy

The presence of very low level of serum biomarkers in the early stage of the disease may be challenging in processing for clinical studies. Recently, for the first time, Raman spectroscopy has been used to assay the blood samples for diagnosis of early and late stage ovarian cancer (Paraskevaidi et al., 2018; Peng et al., 2014). Raman Spectroscopy is based on the phenomenon of inelastic light scattering caused when light interact with matter. Shift in energy of the sample's electron to its original level from its excited state is a characteristic of specific biomolecules including proteins, nucleic acids and lipids, which are informative about the sample. Surface-enhanced Raman spectroscopy (SERS) is the enhanced version of Raman spectroscopy that enhances the Raman signal by 10^3^-10^10^ times. It relies on rough metallic surfaces or nanostructures such as silver or gold nanoparticles and exploits the increase in electromagnetic field from oscillations of surface electrons, termed surface plasmons (Fazio et al., 2016). This enable in the detection of molecules even at low concentration (Lyng et al., 2015; Kong et al., 2015). Using Raman spectroscopy to evaluate the diagnosis of early ovarian cancer has achieved a sensitivity of 93 % and specificity of 97 %. Silver nanoparticle (AgNPs) of 100 nm diameter was used in the enhancement method of SERS for early detection of ovarian cancer and this achieved a sensitivity of 80 % sensitivity and specificity of 94 %. In addition, to differentiate between healthy and ovarian cancer cases, the method was assessed in different levels of CA125 to rule out that the detection accuracy was due to CA125 level, and both techniques provided satisfactory results. Both techniques detected five spectral biomarkers, which are promising as multi-biomarker panel in diagnosing ovarian cancer (Paraskevaidi et al., 2018). These studies highlight the promising role of Raman spectroscopy in the biomarker studies and its adoption in multibiomarker studies will be prospective breakthrough in the field of biomarker research.

### 4.3 Alternative diagnostic approaches

Recent studies also reported the possibilities of autoantibodies, microRNAs and circulating tumor DNA in the blood or fluid from the cervix, uterus or the fallopian tube that could augment the early diagnosis of ovarian cancer in addition to detection of CA125 biomarker (Elias et al., 2018). Microvesicle proteomics of utero-tubal lavage (UtL) liquid biopsies is a contest over serum biomarkers presenting with high sensitivity and specificity in detecting early stage ovarian cancer. Lineage marker such as CA125 demonstrated no significant difference between patients and controls in microvesicle proteomics. However, a 9-protein classifier presented 70 % sensitivity and 76.2 % specificity for diagnosis of high grade ovarian cancer. The use of MS-based targeted assays in the study promises the MS-based clinical tests in increasing the accuracy and multiplexing capabilities in comparison to the commonly used antibody test like ELISA (Barnabas et al., 2019). In addition, the proteomic profiling of tumor fluid of ovarian cancer by LC-MS/MS has been reported as a rich source of proteins to differentiate between the benign and the malignant ovarian tumor (Poersch et al., 2016). Paraneoplastic syndromes are a rare heterogeneous disorders accompanied with the secretion of tumor hormones or autoimmune response caused by tumor cells against onconeural antigens of nervous system or the underlying tumor (Pelosof and Gerber, 2010). Several evidences have reported the presence of different onconeural antibodies in ovarian cancer (Chatterjee et al., 2017). The early onset of paraneoplastic symptoms before the clinical manifestation of cancer appeals its significance and the application of paraneoplastic antigens as biomarkers for early detection of ovarian cancers remain to be explored.

Recently, Skubitz and group (Skubitz et al., 2019) has studied on multi-biomarkers that can differentiate between the serum samples of late stage high grade serous ovarian cancer and healthy control based on Proseek® Multiplex Oncology II plates. This plate can simultaneously measure the expression of 92 cancer related serum based protein biomarkers using proximity extension assays (PEA) (Assarsson et al., 2014; Thorsen et al., 2013; Chen et al., 2015). PEA technology uses a microliter of serum to detect and quantify multiple biomarker that rely on the specificity detection of antibody detection method, and the polymerase chain reaction sensitivity, with accuracy similar to other multiplex detection methods. This study enable in the identification of protein signatures that could differentiate between the healthy individual and the diseased ovarian cancer. From OlinkProseek® Oncology II multiplex assay, 12 proteins were found significantly elevated in ovarian cancer samples, out of which four were newly identified as higher expressing than control. In addition, to meet the minimum screening criteria of 99.6 % specificity, CA125 achieved a sensitivity of 85 %. Further, five biomarkers including FGFBP1, S100A4, EGF, ICOSLG, and MSLN, which were otherwise found at lower level with respect to control, on combination with CA125 enhances the sensitivity and specificity (Skubitz et al., 2019). This perhaps supports the inverse correlation study of additional biomarkers to primary markers (Pinsky and Zhu, 2011). Although this studies relied on serum samples of late stage serous ovarian carcinoma, future biomarker studies based on such Proseek® plates targeting ovarian cancer multi-biomarker will benefit in diagnosing the early stage ovarian cancer. Moreover, to accomplish the highest sensitivity and specificity of early diagnosis of ovarian cancer from an increasing number of promising biomarkers, contribution from different fields of mathematics, computation, physics, chemistry or medicine is needed to develop a prototype to improve the detection efficiency of multi-biomarkers in the clinical settings.

## 5 Conclusion and future perspectives

In the past years, several studies have reported a wide spectrum of serological biomarkers in various combinations. However, reliable validated biomarker(s) in context to specificity, sensitivity and stability, is currently unavailable. Therefore, an optimal multiple biomarkers that improve the early detection with high accuracy are in urgent need.

Following points are highlighted as the key challenges for the early detection biomarker development of ovarian cancer.

• In view of the growing health concerns regarding biomarker development for early detection of ovarian cancer, a wider cohort of high risk ovarian cancer individuals may help in the identification of the best biomarker panels in improving the sensitivity of the biomarkers and in achieving the required sensitivity, specificity and accurate positive predictive value. The responsive collaboration from different research units will help in achieving this feat of selecting the suitable biomarker panels.
• Aptamers targeting ovarian cancer specific unique biomarkers might be a new hope for diagnosis and therapy.
• Multivariate analysis featuring tumor biomarkers along with ultrasound and suitable algorithm will be an added benefit.
• Combination of serum biomarkers with nucleic acid including free DNA, mRNA, microRNAs, and circulating tumor DNA (ctDNA) is also emerging as a diagnostic tool of malignancy (Schwarzenbach et al., 2011; Bettegowda et al., 2014). A matching combination of protein and nucleic acid markers can be a potential tool in the non-invasive screening and diagnosis of ovarian cancer.
• Gene wide transcriptomic profiling may help to determine the aberrant genes to identify the novel biomarkers of ovarian cancer.
• Development of an inexpensive, reliable, robust and quick detection multiplexed biosensor system as a point-of-care diagnostic tool might help in the early diagnosis and timely intervention as major difficulty comes in utilizing circulating biomarkers which are very less in quantity at the early stage of cancer.

## Declarations

### Author contribution statement

All authors listed have significantly contributed to the development and the writing of this article.

### Funding statement

This work was supported by SERB-Department of Science & Technology (SERB-DST), Government of India for providing the Ramanujan Fellowship (SB/S2/RJN087/2014) to MP; and CSIR/UGC JRF, India (21/06/2015(i) EU-V to TM.

### Competing interest statement

The authors declare no conflict of interest.

### Additional information

No additional information is available for this paper.
